# Magnesium Deficiency Alters Expression of Genes Critical for Muscle Magnesium Homeostasis and Physiology in Mice

**DOI:** 10.3390/nu13072169

**Published:** 2021-06-24

**Authors:** Dominique Bayle, Cécile Coudy-Gandilhon, Marine Gueugneau, Sara Castiglioni, Monica Zocchi, Magdalena Maj-Zurawska, Adriana Palinska-Saadi, André Mazur, Daniel Béchet, Jeanette A. Maier

**Affiliations:** 1 UNH, Unité de Nutrition Humaine, Université Clermont Auvergne, INRAE, F-63000 Clermont-Ferrand, France; dominique.bayle@inrae.fr (D.B.); cecile.coudy-gandilhon@inrae.fr (C.C.-G.); marine.gueugneau@inrae.fr (M.G.); andre.mazur@inrae.fr (A.M.); 2 Department of Biomedical and Clinical Sciences Luigi Sacco, Università di Milano, 20157 Milano, Italy; sara.castiglioni@unimi.it (S.C.); monica.zocchi@unimi.it (M.Z.); jeanette.maier@unimi.it (J.A.M.); 3 Biological and Chemical Research Centre, University of Warsaw, PL-02-089 Warsaw, Poland; mmajzur@chem.uw.edu.pl (M.M.-Z.); adusp@cnbc.uw.edu.pl (A.P.-S.); 4 Faculty of Chemistry, University of Warsaw, PL-02-093 Warsaw, Poland; 5 Interdisciplinary Centre for Nanostructured Materials and Interfaces (CIMaINa), Università di Milano, 20133 Milano, Italy

**Keywords:** skeletal muscle, magnesium, magnesium transporters, transcriptome

## Abstract

Chronic Mg^2+^ deficiency is the underlying cause of a broad range of health dysfunctions. As 25% of body Mg^2+^ is located in the skeletal muscle, Mg^2+^ transport and homeostasis systems (MgTHs) in the muscle are critical for whole-body Mg^2+^ homeostasis. In the present study, we assessed whether Mg^2+^ deficiency alters muscle fiber characteristics and major pathways regulating muscle physiology. C57BL/6J mice received either a control, mildly, or severely Mg^2+^-deficient diet (0.1%; 0.01%; and 0.003% Mg^2+^ wt/wt, respectively) for 14 days. Mg^2+^ deficiency slightly decreased body weight gain and muscle Mg^2+^ concentrations but was not associated with detectable variations in gastrocnemius muscle weight, fiber morphometry, and capillarization. Nonetheless, muscles exhibited decreased expression of several MgTHs (*MagT1*, *CNNM2*, *CNNM4*, and *TRPM6*). Moreover, TaqMan low-density array (TLDA) analyses further revealed that, before the emergence of major muscle dysfunctions, even a mild Mg^2+^ deficiency was sufficient to alter the expression of genes critical for muscle physiology, including energy metabolism, muscle regeneration, proteostasis, mitochondrial dynamics, and excitation–contraction coupling.

## 1. Introduction

Magnesium (Mg^2+^) intake is suboptimal in the population of Western countries, which results in an increased risk of latent Mg^2+^ deficiency with Western diet behavior [1]. In addition, there is an increased risk of low Mg^2+^ status in the elderly and after several current pharmacological treatments, such as proton pump inhibitors, thiazides, cetuximab, cisplatin, and some antibiotics [2,3]. In hospitalized patients, hypomagnesemia is a frequent finding often associated with other electrolyte disorders [4]. Clinical manifestations, depending on the severity and chronicity of deficiency, include a large variety of symptoms, e.g., neuromuscular symptoms (hyperexcitability, tetany, cramps, fasciculation, tremor, spasms, weakness), fatigue, tachycardia, anorexia, apathy, and behavioral alterations. In comparison to severe acute Mg^2+^ deficiency, the diagnosis of chronic latent Mg^2+^ deficiency is difficult, because magnesemia is often within reference intervals and results in nonspecific clinical symptoms [5,6]. However, it is well recognized that chronic Mg^2+^ deficiency contributes to a broad range of metabolic, cardiovascular, immune, and neurological disorders [7]. 

Mg^2+^ is the second (after K^+^) most abundant intracellular cation and Mg^2+^ is critical for a number of biological processes. Mg^2+^ is a natural Ca^2+^ antagonist, the activator of more than 200 enzymes, and the direct cofactor of over 600 enzymes [7]. In addition, intracellular Mg^2+^ is buffered by many biological molecules, including proteins, RNAs, DNA, and ATP. ATP is mainly bound to Mg^2+^, and MgATP^2-^ is the active species in enzyme binding and energy production. Therefore, the intracellular concentration of Mg^2+^ must be tightly regulated. This is achieved through the activity of Mg^2+^ permeable channels and transporters. The last few years have seen rapid progress in the identification and characterization of Mg^2+^ transport and homeostasis systems (MgTHs), including transient receptor potential cation channel subfamily M member 6 (TRPM6) and 7 (TRPM7), magnesium transporter 1 (MagT1), magnesium transporter MRS2, solute carrier family 41 member 1 (Slc41a1) and 3 (Slc41a3), cyclin, CBS domain divalent metal cation transport mediator 1 (CNNM1) and 4 (CNNM4) [7,8,9]. Although the precise function of MgTHs is still under investigation, current knowledge suggests that cellular Mg^2+^ homeostasis is regulated by the combined action of several ubiquitous Mg^2+^ transporters. 

Besides its role in energy production, in the skeletal muscle Mg^2+^ controls contraction by acting as a Ca^2+^ antagonist on Ca^2+^-permeable channels and Ca^2+^-binding proteins. Accordingly, Mg^2+^ deficiency decreases muscle strength [10]. Moreover, aging, frequently associated with low Mg^2+^ status, is characterized by the gradual decline of muscle mass and performance. About 25% of body Mg^2+^ is located in the skeletal muscle, which indicates that the expression of MgTHs is relevant to whole-body Mg^2+^ homeostasis. Gene and/or protein expression of ubiquitous MgTHs have been demonstrated in the skeletal muscle (http://www.proteinatlas.org/ accessed on 10 April 2021), but their specific functions in this tissue have not been elucidated. Moreover, to our knowledge, few studies on the regulation of MgTHs in skeletal muscle under pathophysiological conditions, including Mg^2+^ status, have been published [11].

We were interested in unveiling the early events occurring in the skeletal muscle in response to a low Mg^2+^-containing diet. The principal aim of this study was to individuate whether and how a short-term Mg^2+^-deficient diet modulates muscle fiber characteristics and cellular pathways critical for muscle physiology.

## 2. Materials and Methods

### 2.1. Animals

The present study (APAFIS#14025-201803121538803) was approved by the Ethics Committee C2EA-02 and was conducted in accordance with the National Research Council Guide for the Care and Use of Laboratory Animals. All animals were maintained in a temperature-controlled room (22 ± 1 °C) with a 12:12 h light:dark cycle and handled according to the recommendations of the Institutional Ethics Committee. Two-month-old male C57BL/6J mice were housed for one week in a standard environment with a control diet (0.1% Mg^2+^ wt/wt). The mice were then randomly divided into three groups, and over the following two weeks, each group (*n* = 12) was fed one of the three following diets: control diet (0.1% Mg^2+^ wt/wt), mildly Mg^2+^-deficient diet (0.01% Mg^2+^ wt/wt), or severely Mg^2+^-deficient diet (0.003% Mg^2+^ wt/wt). The Ca^2+^ content of the diets was 0.4% (wt/wt). All diets were prepared in our laboratory. Distilled water and food were available ad libitum. Quantitative magnetic resonance of live mice was carried out to estimate body fat and lean mass using an EcoMRI-100 analyzer (Echo Medical Systems LLC, Houston, TX, USA). At the end of the experiment, the animals were sacrificed, blood was collected from the heart in heparin-containing tubes, and gastrocnemius muscles were excised. Plasma was obtained by centrifugation (10 min, 3500 rpm, 4 °C) and frozen for later analysis. Muscles were weighed and (i) maintained in RNAlater (Qiagen, Courtaboeuf, France) overnight at 4 °C before extracting RNA, (ii) frozen in isopentane cooled on liquid N_2_ and stored at −80 °C for histology, or (iii) snap-frozen in liquid N_2_ for muscle Mg^2+^ measurements.

### 2.2. Mg^2+^ Analysis

Plasma magnesium was quantified using a Magnesium Calgamite kit according to the manufacturer’s instructions (Biolabo, Maizy, France). Erythrocytes were washed 3 times with a saline solution, hemolyzed in water, and centrifuged. Muscle samples were mineralized in 65% HNO_3_ for 48 h, and then 1.5 mL of deionized H_2_O and 0.8 mL of 18 mol/L NaOH were added. Magnesium analyses were performed on a chemistry analyzer (Indiko Plus, Thermo Fisher Scientific, Vantaa, Finland).

### 2.3. Fiber Morphometry and Capillary Network

Serial cross sections (10 µm thick) were obtained using a cryostat (Microm, Francheville, France) at −25 °C. Myofiber morphometry and capillarization were assessed on cross sections after labeling with anti-laminin-α1 (Sigma, Saint-Quentin-Fallavier, France) and anti-CD31 (M0823 from Dako, Glostrup, Denmark), respectively, and capturing images by a BX-51 microscope (Olympus, Rungis, France) according to [12,13]. On average, 710 ± 48 fibers were analyzed per subject. Fiber cross-sectional area (CSA) and perimeter were determined for each fiber, using the image processing software Visilog-6.9 (Noesis, Gif-sur-Yvette, France) as previously described [12]. A shape factor (perimeter^2^/4π CSA) was calculated, with a value of 1.0 indicating a circle and >1.0 an increasingly elongated ellipse. A mean of 229 ± 13 capillaries was analyzed per subject. Capillary density (CD) was expressed as the number of capillaries counted per square mm. Capillary-to-fiber ratio (C/F) was calculated as the ratio between the number of capillaries and the number of fibers present in the same area [13].

### 2.4. Quantitative Real-Time Polymerase Chain Reaction (qRT-PCR) Analysis

Independent RNA isolations were carried out for each gastrocnemius muscle sample. Total RNAs were extracted using the RNeasy Fibrous Tissue Mini Kit (Qiagen) following the manufacturer’s conditions. RNA concentrations were measured using a NanoDrop ND-1000 (LabTech, Ringmer, UK), and RNA quality was verified by 1% agarose gel electrophoresis. One μg total RNA was used as a template for single-strand cDNA synthesis using High-Capacity cDNA RT Kit (Applied Biosystems, Foster City, CA, USA) in a total volume of 20 µL containing 1 X RT buffer, 4 mM dNTP mix, 1 X random primers, 50 U reverse transcriptase and 20 U RNase inhibitor. The primer sequence is reported in Table 1. The reverse transcription reactions were run as follows: 25 °C for 10 min, 37 °C for 120 min, and 85 °C for 5 s. PCR was carried out in a final volume of 20 µL containing 10 µL Power SYBR Green PCR Master Mix (Applied Biosystems), 0.4 µL of each primer at 10 pmol/µL, and 2 µL of the cDNA solution. qRT-PCR amplification was performed using a CFX96 Real-Time PCR Detection System (Bio-Rad, Marnes-la-Coquette, France) with the following thermal cycler conditions: 15 min at 95 °C, followed by 45 cycles of 15 s at 95 °C, and 1 min at 60 °C. Raw data were analyzed using CFX Maestro (Bio-Rad) and compared by the ΔΔCt method. Results are expressed relative to the housekeeping gene (*Actb*) transcript quantity.

### 2.5. TaqMan Low-Density Array (TLDA)

A total of 500 ng (10 µL) cDNA of each sample was combined with 95 µL of nuclease-free water and 105 µL 2X TaqMan™ Fast Advanced Master Mix (Applied Biosystems) for the quantitative real-time PCR (qPCR) measurements. This mixture was divided equally over two sample-loading ports of the TLDA. The arrays were centrifuged once (1 min, 1300 rpm at room temperature) to equally distribute the sample over the wells. Subsequently, the card was sealed to prevent exchange between wells. qPCR amplification was performed using an Applied Biosystems 7900HT system with the following thermal cycler conditions: 2 min at 50 °C and 10 min at 94.5 °C, followed by 40 cycles of 30 s at 97 °C and 30 s at 59.7 °C. Raw data were analyzed using Sequence Detection System (SDS) Software v2.4 (Applied Biosystems). The expression of β-actin (*Actb*), β-glucuronidase (*Gusb*), and hypoxanthine phosphoribosyltransferase (*Hprt*) were used as controls. The genes analyzed are reported in Table 2.

### 2.6. Western Blot

Gastrocnemius muscles were mechanically shredded in a Potter homogenizer with lysis buffer (50 mM Tris-HCl pH 7.4, 150 mM NaCl, 1% NP-40, 0.25% Na-deoxycholate) containing protease inhibitors. Total proteins were quantified using the Bradford reagent (Sigma-Aldrich, St. Louis, MO, USA). Equal amounts of proteins were separated by SDS–PAGE on 4–20% Mini-PROTEAN TGX Stain-free Gels (Bio-Rad, Hercules, CA, USA) and transferred to nitrocellulose membranes by using Trans-Blot^®^ TurboTM Transfer Pack (Bio-Rad). After blocking with bovine serum albumin (BSA), Western blot analysis was performed using primary antibodies against Myog, Opa1 (BD Biosciences, St. Diego, CA, USA), Gapdh, and Mfn2 (Santa-Cruz Biotechnology, Dallas, TX, USA). The filters were washed and incubated with secondary antibodies conjugated to horseradish peroxidase (Amersham Pharmacia Biotech Italia, Cologno Monzese, Italy) were used. The immunoreactive proteins were detected with ClarityTM Western ECL substrate (Bio-Rad) and images were captured with a ChemiDoc MP Imaging System (Bio-Rad). The nitrocellulose sheets were used as control loading. Densitometry of the bands was performed with the software ImageLab (Bio-Rad). The Western blots shown are representative and the densitometric analysis was performed on three independent experiments.

### 2.7. Statistical Analysis

Data are presented as means ±SE. To determine whether or not data sets were normally distributed, the Shapiro–Wilk and Kolmogorov–Smirnov normality tests were performed. When data were normally distributed, statistical comparisons between groups were performed applying either Student’s *t* test or one-way ANOVA, followed by a Tukey’s post hoc test, as appropriate. Mann–Whitney U tests were performed when data in at least one group were not normally distributed. Correction for multiple testing was performed with R according to [14], and TLDA significance was set at q-value < 0.05. Univariate linear Pearson’s regression was carried out to investigate relationships between MgTHs mRNA levels and body weight gain. Statistical analyses were performed using XLSTAT (Addinsoft, Paris, France), and significance was set at *P* < 0.05.

## 3. Results

### 3.1. Mg^2+^-Deficient Diet Reduces Muscle Mg^2+^ Concentrations but Does Not Affect Fiber Characteristics

**Figure 1:**
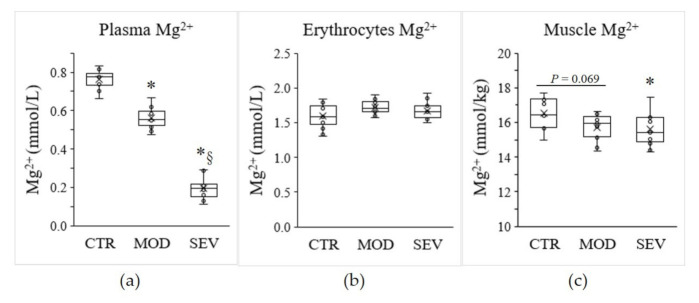
Mg^2+^ status in mice. Mg^2+^ concentrations were measured in (**a**) plasma, (**b**) erythrocytes, and (**c**) gastrocnemius muscle of mice fed either a control (CTR), a mildly (MOD), or a severely (SEV) Mg^2+^-deficient diet. Data (N = 12 per group) are presented as box-and-whisker plots (centerline, median; box limits, first and third quartiles; whiskers, 1.5 x interquartile range; points, outliers; x in the box, mean). * Significant difference (*P* < 0.05) from the CTR group. § Significant difference (*P* < 0.05) from the MOD group.

To investigate the modulation of the expression of Mg^2+^ transport and homeostasis systems (MgTHs) in response to Mg^2+^ status, we used a model of C57BL/6J mice receiving a mildly or severely Mg^2+^-deficient diet and compared it to mice under an Mg^2+^-sufficient diet [15,16]. After 14 days of diet, mice fed a mildly or severely Mg^2+^-deficient diet exhibited a 26% and 75% reduction in plasma Mg^2+^ concentration, respectively (Figure 1a). However, no significant change in erythrocyte Mg^2+^ concentration was detected (Figure 1b). In parallel, moderate and severe Mg^2+^ deficiencies resulted in a 4.8% and 5.4% decrease in intramuscular Mg^2+^ concentrations, respectively, compared to the control Mg^2+^-sufficient diet (Figure 1c). 

**Figure 2:**
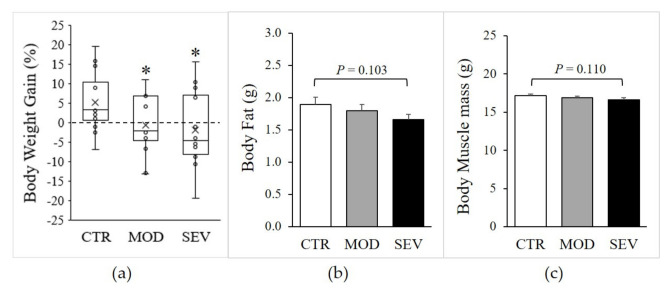
Body composition of mice fed Mg^2+^-deficient diet. Mice were fed either a control (CTR), a mildly (MOD), or a severely (SEV) Mg^2+^-deficient diet: (**a**) body weight, data (N = 12) presented as box-and-whisker plots, (**b**) body fat, and (**c**) body muscle mass were determined by EcoMRI; results are means + SE (N = 12). * Significant difference (*P* < 0.05) from the CTR group.

**Figure 3:**
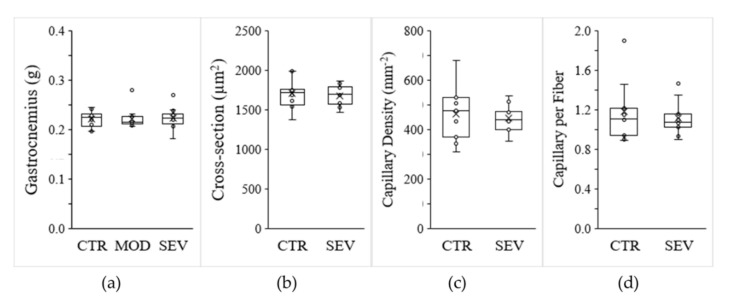
Mg^2+^-deficient diet and muscle characteristics. Mice were fed either a control (CTR), a mildly (MOD), or a severely (SEV) Mg^2+^-deficient diet: (**a**) gastrocnemius muscle weight; (**b**) muscle fiber cross-sectional area; (**c**) muscle capillary density; (**d**) number of capillaries per fiber. Data (N = 12 per group) are presented as box-and-whisker plots.

Moderate and severe Mg^2+^ deficiencies were associated with a significant decline in body weight gain (Figure 2a), and magnetic resonance imaging (EchoMRI) indicated modest trends for whole-body fat and lean mass reductions (Figure 2b,c). Nonetheless, gastrocnemius muscle weight did not differ between Mg^2+^-deficient and Mg^2+^-sufficient diets (Figure 3a). Semiquantitative histology further indicated that Mg^2+^ deficiency was not associated with a detectable variation in muscle fiber cross-sectional area, shape, capillary density (CD), or capillary-to-fiber ratio (C/F) (Figure 3b–d).

### 3.2. Mg^2+^-Deficient Diet Alters Expression of Muscle MgTHs

**Figure 4:**
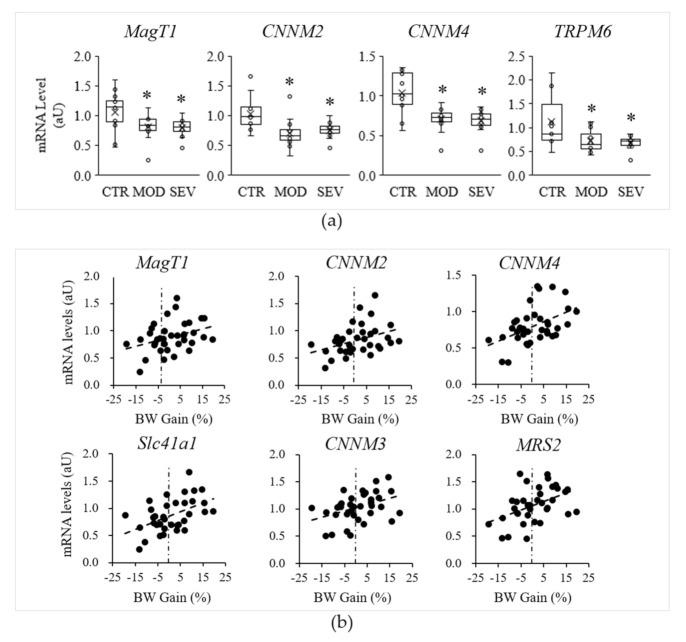
Mg^2+^-deficient diet and muscle Mg^2+^ transport and homeostatic systems (MgTHs) mRNA levels. Mice were fed either a control (CTR), a mildly (MOD), or a severely (SEV) Mg^2+^-deficient diet: (**a**) gastrocnemius MgTHs mRNA levels; data (N = 12) are presented as box-and-whisker plots; * indicates significant difference (*P* < 0.05) from the CTR group; (**b**) examples of linear Pearson’s regressions between muscle MgTHs mRNA levels and body weight (BW) gain.

Although no evidence emerged in terms of atrophy or altered morphology or capillarization of muscle fibers, modest declines in muscular Mg^2+^ concentrations might nonetheless modulate specific mechanisms to maintain cellular Mg^2+^ homeostasis. We assessed whether Mg^2+^ deficiency could be associated with differential expression of MgTHs. qRT-PCR analyses performed on gastrocnemius muscle revealed significant declines in *MagT1*, *CNNM2*, *CNNM4*, and *TRPM6* mRNAs in mice under Mg^2+^-deficient diets, compared to Mg^2+^-sufficient diets (Figure 4a). MagT1 is a controversial protein initially isolated as a critical mediator of Mg^2+^ homeostasis in eukaryotes [17] and then as an integral part of the *N*-linked glycosylation complex [18]. As MagT1 mutations associate with hypomagnesemia, we included MagT1 in the list of MgTHs. The mRNA levels of the other MgTHs, i.e., *CNNM1*, *CNNM3*, *MRS2*, *Slc41a1*, *Slc41a2*, and *Slc41a3*, were not altered by Mg^2+^ deficiency. 

Interestingly, regression analyses performed with all mice indicated positive correlation between body weight gain and several MgTHs mRNAs, i.e., *MagT1* (r = 0.35, *P* = 0.038), *CNNM2* (r = 0.38, *P* = 0.022), *CNNM3* (r = 0.40, *P* = 0.015), *CNNM4* (r = 0.48, *P* = 0.003), *MRS2* (r = 0.45, *P* = 0.006), *Slc41a1* (r = 0.50, *P* = 0.002) (Figure 4b).

### 3.3. Mild Mg^2+^ Deficiency Alters the Expression of Genes Important for Muscle Energy Metabolism and Regeneration 

**Figure 5:**
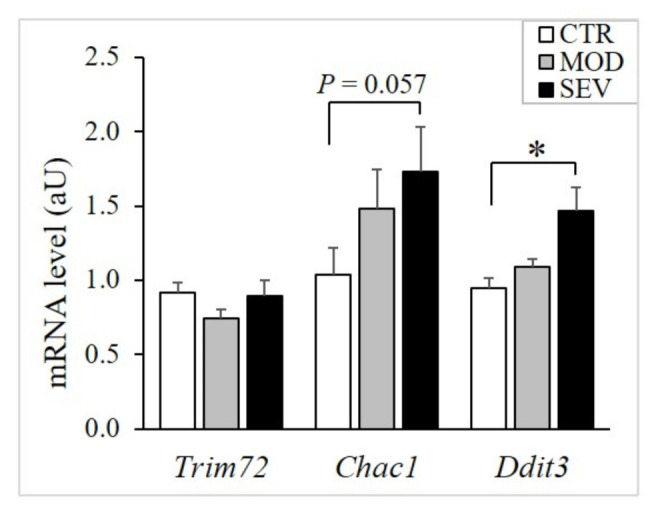
Muscle mRNA levels of stress genes in mice fed Mg^2+^-deficient diet. TaqMan low-density array (TLDA) was used to measure mRNA levels in gastrocnemius muscle of mice fed either a control (CTR; white bars), a mildly (MOD; grey bars), or a severely Mg^2+^-deficient diet (SEV; black bars). * indicates significant difference (*P* < 0.05) from the CTR group.

As Mg^2+^ is the activator or cofactor of a number of enzymes, modest declines in intramuscular Mg^2+^ concentrations might modulate biological processes that are critical for muscle physiology. Initially, to assess muscle stress, we evaluated the expression of *Trim72*, *Chac1*, and *Ddit3*. *Trim72* codes for a protein specifically located in the sarcolemma and involved in membrane repair [19]. *Chac1* encodes a protein acting downstream of ATF4 implicated in muscle atrophy [20], and *Ddit3* encodes a member of the CCAAT/enhancer-binding protein (C/EBP) family of transcription factors and inhibits myogenesis [21]. We found that severely and mildly Mg^2+^-deficient diets did not modulate *Trim72* (Figure 5). Moreover, severe Mg^2+^ deficiency, but not mild Mg^2+^ deficiency, upregulated the stress genes *Chac1* and *Ddit3* (Figure 5). 

**Figure 6:**
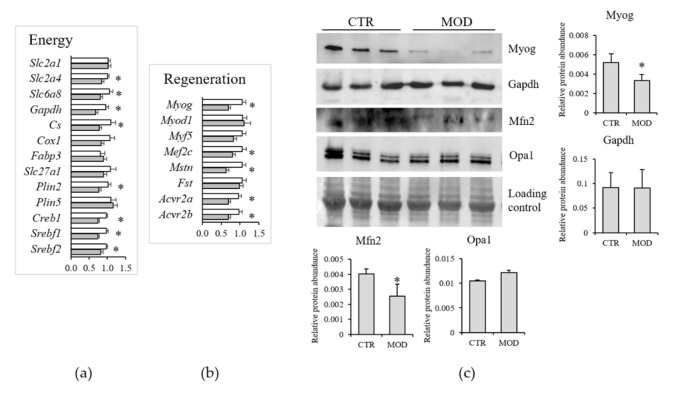
Muscle mRNA levels of genes involved in energy metabolism (**a**) and muscle regeneration (**b**) in mice fed a mildly Mg^2+^-deficient diet. Results are means ± SE (*N* = 12). White columns CTR, grey columns mild Mg^2+^ deficiency. (**c**) Western blot was performed on 40 µg of lysates using specific antibodies against Myog, Gapdh, Mfn1, and Opa1. A representative western is shown. Densitometry was performed on three different blots. * indicates significant difference (*P* < 0.05) from the CTR group.

To reduce the potential interferences due to the activation of the stress response and to mimic chronic latent deficiency conditions, further investigations were focused on mild Mg^2+^ deficiency. Moreover, TaqMan low-density array (TLDA) analyses revealed that mild Mg^2+^ deficiency was sufficient to rapidly alter the expression of genes important for lipid and carbohydrate metabolism (Figure 6a). These included *Slc2a4* and *Slc6a8*, coding for Glut4 and creatine transporter (CT)-1, respectively, *Gapdh*, *Cs* (citrate synthase), *Plin2*, a marker of intramyocellular lipid droplets, and the transcription factors *Creb1*, *Srebf1*, and *Srebf2*. Mild Mg^2+^ deficiency also rapidly altered the expression of genes involved in muscle regeneration (Figure 6b), i.e., *Myog* (myogenin), *Mef2c* (myocyte enhancer factor 2C), *Mstn* (myostatin), and its receptors (*Acvr2a* and *Acvr2b*). 

We tested the total amounts of some proteins by Western blot on lysates from gastrocnemius muscles of 12 animals under control or mildly Mg^2+^-deficient diet. As shown in Figure 6c, we found a significant reduction of Myog and no modulation of Gapdh.

### 3.4. Mild Mg^2+^ Deficiency Alters Expression of Genes Important for Muscle Proteostasis

**Figure 7:**
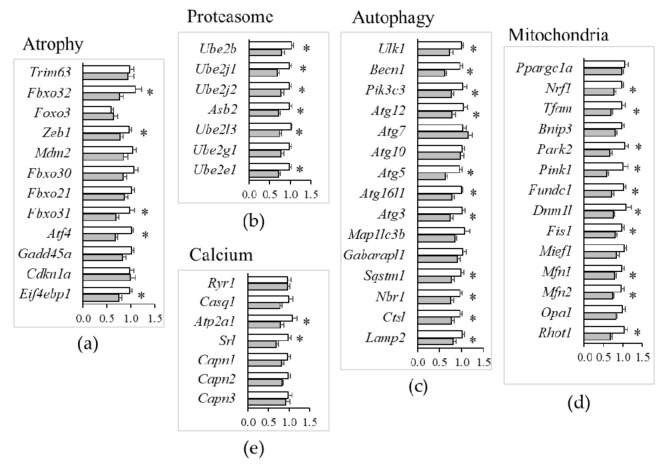
Muscle gene expression in mice fed a mildly Mg^2+^-deficient diet. TaqMan low-density array (TLDA) was used to measure mRNA levels of genes critical for muscle physiology of mice fed either a control (white bars) or a mildly (grey bars) Mg^2+^-deficient diet. Genes involved in muscle atrophy (**a**), proteostasis (**b**), autophagy (**c**), mitochondrial dynamics (**d**) and calcium homeostasis (**e**) were analyzed. Results are means ± SE (N = 12). * indicates significant difference (*P* < 0.05) from the CTR group.

Amongst the transduction pathways implicated in the regulation of muscle atrophy [22,23], mild Mg^2+^ deficiency was associated with a decreased expression of *Fbxo32* (MAFbx), *Zeb1*, *Fbxo31*, *Atf4*, and *Eif4ebp1*, while the mRNA levels of other major regulators, e.g., *Trim63* (MuRF1), *Foxo3*, *Mdm2*, *Fbxo30* (Musa1), *Fbxo21* (Smart), *Gadd45a*, and *Cdkn1a* (p21) did not significantly change (Figure 7a). 

In agreement with the downregulation of major atrogenes by mild Mg^2+^ deficiency, e.g., *Fbxo32* (MAFbx), there was also evidence for altered expression of many genes involved in protein catabolism. This encompassed the downregulation of (i) E2-activating genes (*Ube2b*, *Ube2j1*, *Ube2j2*, *Asb2*, *Ube2l3*, *Ube2e1*) of the ubiquitin–proteasome system (Figure 7b); (ii) genes regulating initiation (*Ulk1*, *Becn1*, *Pik3c3*), elongation (*Atg12*, *Atg5*, *Atg16l1*, *Atg3*), substrate/cargo recruitment (*Sqstm1*, *Nbr1*) and lysosomal proteolysis (*Ctsl*, *Lamp2*) in autophagy (Figure 7c). However, Mg^2+^ deficiency did not affect the expression of Ca^2+^-dependent proteases (*Capn1*, *Capn2*, *Capn3* calpains) (Figure 7e).

### 3.5. Mild Mg^2+^ Deficiency, Mitochondria, and Ca^2+^ Homeostasis

Mitochondria play multifaceted roles in essential aspects of skeletal muscle cell physiology [24]. Our TLDA analyses indicated that a mild Mg^2+^ deficiency is sufficient to downregulate genes regulating mitophagy (*Park2*, *Pink1*, *Fundc1*) (Figure 7d), in addition to reducing the expression of genes implicated in mitogenesis (*Nrf1*, *Tfam*), mitochondrial fission (*Dnm1l*, *Fis1*), and fusion (*Mfn1*, *Mfn2*, *Rhot1*) (Figure 7d). By Western blot, we confirmed the downregulation of mitofusin (Mfn)2 (Figure 6c). Opa1, instead, was not modulated, both at the RNA and the protein levels (Figure 6c and Figure 7d).

Mg^2+^ may also modulate Ca^2+^-permeable channels and Ca^2+^-binding proteins. Ca^2+^ handling by the sarcoplasmic reticulum is a key feature in muscle contraction. Action potentials elicit contraction by the release of Ca^2+^ from the sarcoplasmic reticulum through the ryanodine receptors (*Ryr1*) that are regulated by calsequestrin-1 (*Casq1*). For muscle relaxation, Ca^2+^ is transported back to the sarcoplasmic reticulum by sarco/endoplasmic reticulum Ca^2+^-ATPases (Serca), regulated by sarcalumenins (*Srl*) [25,26]. It is noteworthy that our TLDA study emphasized that a mild Mg^2+^ deficiency did not affect the expression of genes involved in contraction (*Ryr1*, *Casq1*), while it downregulated the expression of those implicated in relaxation, *Atp2a1* (Serca), and *Srl* (Figure 7e).

## 4. Discussion

Appropriate nutrition is indispensable for normal muscle metabolism and function [7]. In humans, cross-sectional associations between low Mg^2+^ intake and loss of skeletal mass and function were reported in large population cohorts [27,28,29].

To get insight into the role of Mg^2+^ in muscle health, we developed an experimental model in which mice were fed a moderately or severely Mg^2+^-deficient diet. Both these dietetic regimens significantly reduced serum Mg^2+^ levels, a useful biomarker of Mg^2+^ status [6]. Unexpectedly, we did not detect a reduction of erythrocytes Mg^2+^ concentration, differently from a previous report demonstrating the concomitant reduction of serum and erythrocyte Mg^2^ in animals fed the severely deficient diet [15]. We have no explanation at the moment for this discrepancy. Interestingly, we found that some Mg^2+^ transporters were significantly downregulated in the muscle. This finding explains the reduced amounts of intracellular Mg^2+^ in the muscle and might also represent an initial, adaptive response aimed at maintaining circulating Mg^2+^ as close to physiological levels as possible. In this perspective, it is noteworthy that 12–13 week-old *Trpm6*-deficient adult mice are hypomagnesemic and sarcopenic, events reversible upon supplementation with Mg^2+^ [30]. Additionally, *CNNM*2+/− mice were hypomagnesemic, but no data are available on the skeletal muscle yet [31]. In our experimental model, Mg^2+^ deficiency was associated with a significant decline in body weight gain. Interestingly, regression analysis revealed a positive correlation between body weight gain and the downregulation of *MagT1*, *CNNM2*, *CNNM3*, *CNNM4*, *MRS2*, *Slc41a1* in the muscle. Indeed, redundancy among members of Mg^2+^ transporters likely enables functional compensation to maintain sufficient Mg^2+^ homeostasis resulting in normal body weight.

We found no structural signs of muscle atrophy after 14 days in Mg^2+^ deficient regimen. This finding might be due to the downregulation of the genes coding for myostatin and its receptors, which activate the ubiquitin–proteasome and autophagy pathways, thus resulting in muscle wasting [32]. Accordingly, TLDA analysis revealed the reduced expression of genes involved in the regulation of the proteasome and autophagy in mice fed a moderately low Mg^2+^ diet.

By TLDA analysis, we found significant differences in the expression of genes coding for proteins involved in energy metabolisms. Glucose transport into cells is the first step in glucose metabolism. We here describe the downregulation of the gene encoding the glucose transporter Glut4. Interestingly, in the gastrocnemius of type 2 diabetic rats, Glut4 was reduced, and Mg^2+^ supplementation was sufficient to revert it [33]. The low expression of Glut4 in Mg^2+^-deficient mice indicates that less glucose might be available for energy production, further impaired by the downregulation of *Citrate synthase*, involved in the first reaction of the Krebs cycle. Reduced amounts of *Plin2*, *Srebf1,* and *Srebf2* transcripts might be predictive of altered lipid metabolism. It is noteworthy that *Srebf1* and *Srebf2* are downregulated in the skeletal muscle of diabetic individuals [34] and that the overexpression of *Plin2* ameliorates insulin sensitivity in skeletal muscle [35]. Additionally, *Slc6a8*, coding for creatine transporter (CT)-1, is reduced in moderately Mg^2+^-deficient mice. In the skeletal muscle, the creatine system is fundamental for optimal energy utilization, especially at the beginning of exercise and during intense physical activity, because it serves as a first-line energy buffer that maintains ATP levels constant. CT1 is the major route for creatine entry in skeletal muscle cells and has a central role in ensuring high intracellular creatine content [36]. Consistently, CT1-deficient mice feature muscle atrophy, reduced strength, and endurance [37].

TLDA also disclosed the reduced expression of genes involved in mitophagy, fusion, and fission, thus indicating alterations of mitochondrial dynamics that might be accompanied by impaired energy production in Mg^2+^-deficient mice. Moreover, we found a marked reduction of the total amounts of Mfn2, a GTPase located on the outer mitochondrial membrane which is critical for mitochondrial fusion [38]. It is noteworthy that altered mitochondria have been implicated in sarcopenia in the elderly, in muscle atrophy associated with disuse, in muscular dystrophies, and in insulin resistance [24]. TLDA also shows the downregulation of genes that coordinate the different steps of autophagy in Mg^2+^-deficient mice. This result is in agreement with data from cultured cells. Indeed, both *TRPM7* or *MagT1* silencing and Mg^2+^ deficiency activate autophagy in human mesenchymal stem cells induced osteogenic differentiation [39]. Consistently, high concentrations of extracellular Mg^2+^ inhibit autophagy in chondrocyte ATDC5 cells [40].

Our study presents some limitations. First of all, the experiments were performed using young growing animals. It will be relevant to extend our studies using animals of different ages and to evaluate metabolic parameters related to glucose metabolism and lipid metabolism to obtain a complete overview of the fundamental function exerted by skeletal muscle. We also highlight that our study was performed on male mice. To identify potential gender-related differences, the same experiments should be performed on females. Another limitation is that only some proteins were evaluated. Further experiments are required to confirm the altered expressions at the protein level and/or their functional activity.

## 5. Conclusions

Our results emphasize that even a mild Mg^2+^ deficiency, as found in the Western population, is sufficient to modulate the gene expression of major pathways, mostly related to energy metabolism, proteostasis, autophagy, and mitochondrial dynamics in the skeletal muscle. Consequently, supplementing Mg^2+^ in Mg^2+^-deficient individuals might be a simple and costless countermeasure to maintain healthy muscles and metabolic balance.

## Figures and Tables

**Table 1:** Sequence of primers used for qRT-PCR.

Primers	Forward	Reverse
*TRPM6*	GACAGTCTAAGCACCTTTTC	AAGCTTGTACCCTTCAGTAG
*MagT1*	GATGGGCTTTTGCAGCTTTGT	GCAATACACATCATCCTTCGCT
*MRS2*	GGTGATGTGCTCCGGTTTAGA	TGGCCTGGAGTGCTAACTCAT
*Slc41a1*	TACTGGCCCTACTCCTTCTCC	GGGACTCAATCACTACCACCTC
*Slc41a2*	CTTCAGCAAGAGATCAGAGCC	CCAGCATAGTCATCGTACTTGG
*Slc41a3*	CTCAGCCTTGAGTTCCGCTTT	GCAGGATAGGTATGGCGACC
*CNNM1*	GTAGGGTCACAACCTACATC	CATGACATACACAGAAGAGG
*CNNM2*	AAGTGGCCCACCGTGAAAG	CGCTTCTACTTCTGTTGCTAGG
*CNNM3*	GACTCCGGCACTGTCCTAGA	AGTGGATGGTTGTAGAAGCGG
*CNNM4*	CTGCACATCCTTCTCGTTATGG	TGCGAGCATACTTTCTCTCCTT

**Table 2:** Gene analyzed.

***Acvr2a***	Activin A Receptor Type 2a	***Map1lc3b***	Microtubule-Associated Protein 1 Light Chain 3 Beta
***Acvr2b***	Activin A Receptor Type 2b	***Mdm2***	Mouse double minute 2 homolog
***Asb2***	Ankyrin Repeat And SOCS Box Containing 2	***Mef2c***	Myocyte Enhancer Factor 2C
***Atf4***	Activating Transcription Factor 4	***Mfn1***	Mitofusin 1
***Atg10***	Autophagy Related 10	***Mfn2***	Mitofusin 2
***Atg12***	Autophagy Related 12	***Mief1***	Mitochondrial Elongation Factor 1
***Atg16l1***	Autophagy Related 16 Like 1	***Mstn***	Myostatin
***Atg3***	Autophagy Related 3	***Myf5***	Myogenic Factor 5
***Atg5***	Autophagy Related 5	***Myod***	Myogenic Differentiation 1
***Atg7***	Autophagy Related 7	***Myog***	Myogenin
***Atp2a1***	ATPase Sarcoplasmic/Endoplasmic Reticulum Ca^2+^ Transporting 1	***Nbr1***	NBR1 Autophagy Cargo Receptor
***Becn1***	Beclin 1	***Nrf1***	Nuclear Respiratory Factor 1
***Bnip3***	BCL2/adenovirus E1B Interacting Protein 3	***Opa1***	Mitochondrial dynamin like GTPase
***Capn1***	Calpain 1	***Park2***	Parkin RBR E3 Ubiquitin Protein Ligase
***Capn2***	Calpain 2	***Pik3c3***	Phosphatidylinositol 3-Kinase Catalytic Subunit Type 3
***Capn3***	Calpain 3	***Pink1***	PTEN Induced Kinase 1
***Casq1***	Calsequestrin 1	***Plin2***	Perilipin 2
***Cdkn1a***	Cyclin Dependent Kinase Inhibitor 1A	***Plin5***	Perilipin 5
***Chac1***	Glutathione-specific gamma-glutamylcyclotransferase 1	***Ppargc1a***	Peroxisome Proliferative Activated Receptor Gamma Coactivator 1 Alpha
***CNNM2***	Cyclin and CBS Domain Divalent Metal Cation Transport Mediator 2	***Rhot1***	Ras Homolog Family Member T1
***CNNM4***	Cyclin and CBS Domain Divalent Metal Cation Transport Mediator 4	***Ryr1***	Ryanodine Receptor 1
***Cox1***	Mitochondrially Encoded Cytochrome C Oxidase I	***Slc27a1***	Solute Carrier Family 27 Member 1
***Creb1***	CAMP Responsive Element Binding Protein 1	***Slc2a1***	Solute Carrier Family 2 Member 1
***Cs***	citrate synthase	***Slc2a4***	Solute Carrier Family 2 Member 4
***Ctsl***	Cathepsin L	***Slc6a8***	Solute Carrier Family 6 Member 8
***Ddit3***	DNA Damage Inducible Transcript 3	***Slc41a1***	Solute Carrier family 41 member 1
***Dnm1l***	Dynamin 1 Like	***Sqstm1***	Sequestosome 1
***Eif4ebp1***	Eukaryotic Translation Initiation Factor 4E Binding Protein 1	***Srebf1***	Sterol Regulatory Element Binding Transcription Factor 1
***Fabp3***	Fatty Acid Binding Protein 3	***Srebf2***	Sterol Regulatory Element Binding Transcription Factor 2
***Fbxo21***	F-Box Protein 21	***Srl***	Sarcalumenin
***Fbxo30***	F-Box Protein 30	***Tfam***	Transcription Factor A, Mitochondrial
***Fbxo31***	F-Box Protein 31	***Trim63***	Tripartite Motif-containing 63
***Fbxo32***	F-Box Protein 32	***Trim72***	Tripartite Motif-containing 72
***Fis1***	Fission, Mitochondrial 1	***TRPM6***	Transient Receptor Potential cation channel subfamily M member 6
***Foxo3***	Forkhead Box O3	***Ube2b***	Ubiquitin Conjugating Enzyme E2 B
***Fst***	Follistatin	***Ube2e1***	Ubiquitin-Conjugating Enzyme E2 E1
***Fundc1***	FUN14 Domain Containing 1	***Ube2g1***	Ubiquitin-Conjugating Enzyme E2G 1
***Gabarapl1v***	Gamma-Aminobutyric Acid A Receptor-Associated Protein-Like 1	***Ube2j1***	Ubiquitin-Conjugating Enzyme E2J 1
***Gadd45a***	Growth Arrest and DNA Damage Inducible Alpha	***Ube2j2***	Ubiquitin-Conjugating Enzyme E2J 2
***Gapdh***	Glyceraldehyde-3-Phosphate Dehydrogenase	***Ube2l3***	Ubiquitin-Conjugating Enzyme E2L 3
***Lamp2***	Lysosomal-Associated Membrane Protein 2	***Ulk1***	Unc-51 Like Autophagy Activating Kinase 1
***MagT1***	Magnesium Transporter 1	***Zeb1***	Zinc Finger E-Box Binding Homeobox 1

## Data Availability

The data presented in this study are openly available in INRA Dataverse at https://data.inrae.fr/dataset.xhtml?persistentId=doi:10.15454/IVBODD (accessed on 24 June 2021).
